# Dissection and integration of the autophagy signaling network initiated by bluetongue virus infection: crucial candidates ERK1/2, Akt and AMPK

**DOI:** 10.1038/srep23130

**Published:** 2016-03-15

**Authors:** Shuang Lv, Qing-Yuan Xu, En-Cheng Sun, Ji-Kai Zhang, Dong-Lai Wu

**Affiliations:** 1 State Key Laboratory of Veterinary Biotechnology, Harbin Veterinary Research Institute, Chinese Academy of Agricultural Sciences, Harbin 150001, China

## Abstract

Bluetongue virus (BTV), a complex double-stranded segmented RNA virus, has been found to initiate cellular autophagy for its own benefit. Here, with a view to understanding the underlying mechanisms, we first systematically dissected the exact signaling network in BTV-induced autophagy. We found that the activity of mTOR, a crucial pivot, was inhibited by BTV1 infection, subsequently leading to downstream p70S6K suppression and autophagy initiation. We then explored the upstream regulators of mTOR and analyzed their activities via a series of assays. We found BTV1-induced autophagy to be independent of the ERK1/2 signaling pathway. However, the BTV1-induced inhibition of PI3K/Akt was found to be partially responsible for mTOR inactivation and subsequent autophagy initiation. Furthermore, we found unexpectedly that AMPK seemed to play a more important role in BTV1-induced autophagy. Elevated [Ca^2+^]_cyto_-mediated activation of CaMKKβ exactly managed the activation of AMPK, which then positively regulated autophagy through suppressing mTOR. We must emphasize that TSC2 is a fatal mediator between upstream Akt or AMPK and downstream mTOR through its phosphorylation. Taken together, our data suggested that the BTV1-induced inhibition of the Akt-TSC2-mTOR pathway and the upregulation of the AMPK-TSC2-mTOR pathway both contributed to autophagy initiation and further favored virus replication.

Bluetongue virus (BTV), a double-stranded RNA (dsRNA) virus, is a member of the genus *Orbivirus* within the family *Reoviridae*. As an arthropod-borne virus, it is responsible for a hemorrhagic disease affecting domestic animals, primarily sheep and cattle, as well as some wild ruminants^1^,^2^. The impact of bluetongue disease was relatively low before the 1990s; however, several seasonal incursions of distinct BTV serotypes/strains have occurred virtually every year since 1998, causing massive economic losses to animal husbandry^3^,^4^,^5^. Due to its economic significance, BTV has been studied extensively from perspectives of molecular virology and structural biology, and great progress has been made^6^,^7^,^8^. However, research on the interactions between the virus and host remains relatively weak, highlighting the need for in-depth understanding of BTV pathogenicity in the host.

As obligate intracellular parasites, viruses have limited coding capacity, and their survival is extremely reliant on host cells. To understand the pathogenicity of a virus, one of the key questions in virology is how viruses gain temporary or long-term control over their hosts. Viruses must evolve strategies that utilize or usurp cellular processes to facilitate their own propagation. Macroautophagy (hereafter referred to as autophagy) is a conserved cellular catabolic process that mediates the removal of damaged organelles and long-lived cytoplasmic macromolecules via a lysosomal degradative pathway^9^. Recently, a multitude of studies have reported that viral infections have complex interconnections with the autophagic process^10^. Autophagy has intracellular antimicrobial properties and plays a role in immune responses to viral infections. However, many viruses, including a number of RNA viruses, have been shown to evade or subvert this process to benefit their replication^11^,^12^,^13^. A similar phenomenon was also observed in our previous study, which revealed that BTV infection can activate autophagy and utilize it to promote viral replication^14^. However, subsequent corresponding mechanisms underlying these observations have not yet been elucidated.

Autophagy is activated in response to extra- or intracellular stimuli and signals such as starvation, hypoxia, ER stress, and pathogen infection. Many efforts have been made in the past few years to decipher the regulatory network of autophagy initiation from yeast to mammals^15^,^16^,^17^. Several signaling cascades have been reported to regulate autophagy. The major control complex for autophagy is the mammalian target of rapamycin (mTOR), which, when active, inhibits the initiation of the pathway^18^,^19^,^20^. Many diverse cellular signals converge on mTOR complex 1 (mTORC1) to regulate autophagy upstream of the core machinery^21^. The serine/threonine kinase Akt (also known as PKB) has been shown to modulate autophagic activity regulated by the phosphoinositide 3-kinase (PI3K) pathway^22^,^23^. Mutations causing Akt activation or inactivation would, respectively, suppress and induce autophagy. Molecules upstream of mTOR, such as AMP-activated protein kinase (AMPK) and extracellular signal-regulated kinase 1/2 (ERK1/2), have been shown to inhibit mTORC1 and lead to activation of the autophagic process. AMPK, a crucial factor in sensing cellular energy and ion homeostasis^24^,^25^, negatively regulates mTOR either by directly inhibiting mTOR or by activating tuberous sclerosis protein 2 (TSC2) protein, an upstream negative effector of mTOR^26^,^27^,^28^. Mitogen-activated protein kinases (MAPKs) are central molecules mediating signaling pathways in innate immunity. To date, seven distinct groups of MAPKs have been characterized in mammalian cells. Of these seven, the most extensively studied are ERK1/2, c-Jun N-terminal kinases (JNKs) and p38 MAPKs (p38 α/β/γ/δ), which have also been reported to regulate autophagy activity^29^. In addition, endoplasmic reticulum (ER) stress, p53, nuclear factor-κB (NF-κB) and other mTOR-independent pathways are also involved in the regulatory network of autophagy^30^,^31^,^32^.

There are reports that many viruses can activate and exploit certain signaling pathways related to autophagy for more efficient replication, depending on the virus type or stage of viral infection^33^,^34^. However, until now, for BTV, the precise mechanisms and signaling pathways of modulating cellular autophagy have remained largely unknown.

Based on this information, we have undertaken a comprehensive investigation to explore the autophagy signaling network initiated by BTV infection, focusing on these vital and canonical candidates. Our results demonstrate that BTV1 induces autophagy in BSR cells by setting off distinct cascade reactions involving Akt, AMPK, and ERK1/2 to repress central mTOR for its own benefits.

## Results

### BTV1-mediated suppression of mTOR activity is associated with autophagy induction

**Figure 1:**
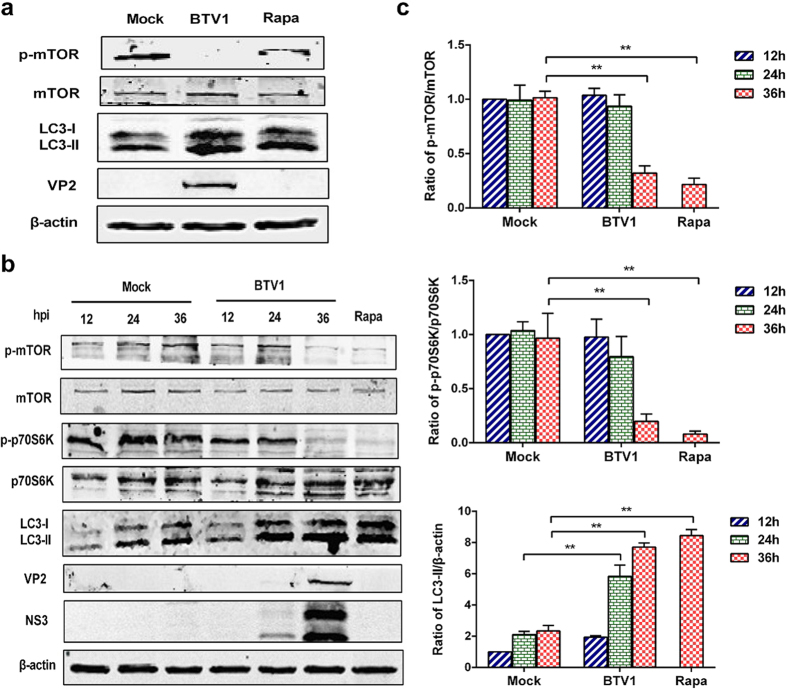
BTV1 induces autophagy by suppressing the mTOR signaling pathway. (**a**) BSR cells were infected with BTV1 (MOI = 1) or mock-infected or treated with 100 nM rapamycin (Rapa, positive control) for 36 h. Western blotting was performed using the indicated antibodies. (**b**) BSR cells were infected as above, and at different time points (12, 24 and 36 hpi), cells were harvested and analyzed by western blotting using the indicated antibodies. BSR cells treated with 100 nM Rapa for 36 h were used as a positive control. (**c**) Bar graphs showed the relative abundances of phosphorylated mTOR (p-mTOR), p-p70S6K and LC3-II protein (normalized to corresponding total protein or β-actin). A mock infection of 12 h was set at 1.0. The values represent the mean ± SD from three repeats. Statistical significances were analyzed using Student’s t-test (**P < 0.01).

Our previous studies demonstrated that BTV1 infection triggered autophagy for its own benefit^14^. To probe the underlying mechanism by which BTV1 induced autophagy, we analyzed the mTOR pathway in BSR cells because mTOR is the major hub of the whole regulatory network of autophagy^18^. Rapamycin, an mTOR inhibitor, was used as a positive control. As shown in Fig. 1a, compared with mock cells, BTV1 infection and rapamycin treatment both caused significant decreases in the levels of phospho-mTOR, while the total mTOR remained nearly unchanged. The ratio of LC3-II to β-actin, a marker of autophagy, clearly increased, indicating that mTOR signaling is involved in BTV1- induced autophagy in BSR cells.

To confirm whether mTOR is repressed by BTV1 in a replication stage-dependent manner, we performed an immunoblotting assay using samples collected at various infection times. The results showed that mTOR phosphorylation activity displayed a reduced trend with the replication of the virus. Consistently, the phosphorylation of ribosomal protein S6 kinase-1(p70S6K), a hallmark substrate of mTORC1, was also found to be diminished with increasing infection time (Fig. 1b). The ratios of p-mTOR/mTOR and p-p70S6K/p70S6K were notably decreased by approximately 3.5 times and 4.5 times, respectively, at 36 hpi. (Fig. 1c, *Upper and middle panels*, *P* < 0.01). As expected, the increase in LC3-II was monitored at the indicated times after BTV1 infection relative to mock-infected cells (Fig. 1b). Similarly, the indicator ratio of LC3-II to β-actin was significantly increased by approximately 3 times at 24 hpi and 4 times at 36 hpi (Fig. 1c, *Lower panel*, *P* < 0.01). Meanwhile, monoclonal antibodies that specifically recognize the BTV1 VP2 and NS3 proteins were used to track the progression of infection. Collectively, these observations indicate that BTV1 induces autophagy by inhibiting mTOR signaling, and the process is dependent on viral replication.

### Autophagy initiated by BTV1 is not dependent on the ERK1/2-mTOR pathway

To investigate the upstream regulators targeting mTOR, a systematic screening strategy was formulated. Because the ERK1/2 pathway is reported to play a key role in the inhibition of mTOR and thus the positive regulation of autophagy^35^, we first examined whether ERK1/2 participates in autophagy induction in BTV1 infected cells. Because the autophagy phenomenon and mTOR activity are more obvious during the middle-late stage of virus replication, the following tests all used two timepoints: 24 and 36 hpi.

**Figure 2:**
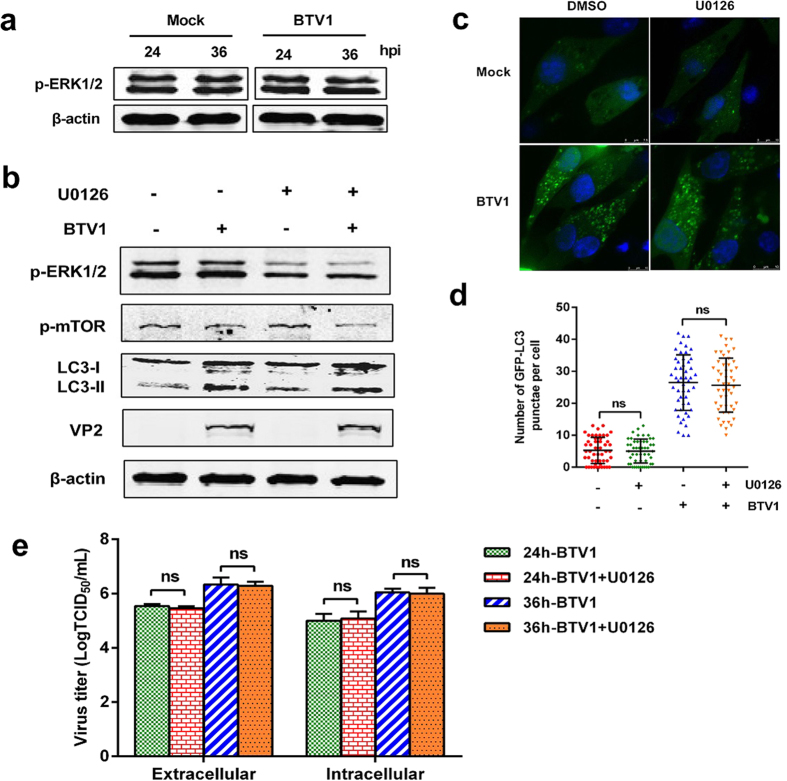
Autophagy initiated by BTV1 is not dependent on the ERK1/2-mTOR pathway. (**a**) BSR cells were infected with BTV1 (MOI = 1) for 24 and 36 h. Cell lysates were analyzed by western blotting for p-ERK1/2 expression. (**b**) Effects of inhibiting ERK1/2 activity by U0126 treatment on endogenous LC3-II, p-ERK1/2 and p-mTOR levels. The levels of related proteins were detected by Western blotting after mock-infection or BTV1-infection in the presence or absence of 20 μM U0126 for 36 h. (**c**) BSR cells were transfected with a plasmid expressing EGFP-LC3. At 12 hpt, the cells were treated as in (**b**). Formation of autophagosomes, shown as green punctae following nuclear DAPI staining and analyzed by confocal fluorescence microscopy (scale bar: 7.5 or 10μm). (**d**) Average number of green punctae per cell from 50 random cells in each treatment. The data of all groups are reported as the mean ± SD (ns, *P* > 0.05; *P < 0.05; and **P < 0.01), as in the subsequent figures. (**e**) The effects of 20 μM U0126 on the production of infectious particles of BTV1. The extracellular and intracellular virus loads were tested as TCID_50_/mL at 24 hpi and 36 hpi, respectively. Data represent the mean ± SD of three independent experiments. Statistical significances were analyzed by two-way ANOVA (*P < 0.05 and **P < 0.01, significantly different), as in the subsequent figures.

Figure 2a shows that the bands of phospho-ERK1/2 hardly changed in BTV1-infected cells relative to the uninfected group, which implied that phospho-ERK1/2 was not involved in BTV1-induced autophagy. Further confirmation assays were conducted by the inhibition of ERK1/2 phosphorylation with the specific inhibitor U0126. The results showed that the p-ERK1/2 band reduced significantly with U0126 treatment, meaning the inhibitory effect of the drug was obvious. However, the inhibition of p-ERK1/2 by U0126 hardly affect the trends of mTOR phosphorylation and LC3 conversion caused by BTV1 infection (Fig. 2b, Lane 2 and 4), which suggested that ERK1/2 was not the functional upstream regulator of mTOR-LC3 pathway in BTV1 infection (Fig. 2b). Additionally, immunofluorescence analysis was used to quantify the LC3 punctae, another marker of autophagy. After transfection with pEGFP-LC3, the addition of U0126 did not obviously alter the number of EGFP-LC3 punctae relative to the DMSO group with or without BTV1 infection in BSR cells (Fig. 2c,d, *P* > 0.05). Thus, we conclude that ERK1/2 signaling is not involved in BTV1-induced autophagy. Our findings of viral titer determination are consistent with previous observations of VP2 in western blotting, indicating that U0126 treatment did not affect virus replication (Fig. 2e). All the above results suggest that BTV1-induced autophagy initiation is not dependent on the ERK1/2-mTOR pathway.

### Down-regulation of p-mTOR is related to Akt inactivation after BTV1 infection

**Figure 3:**
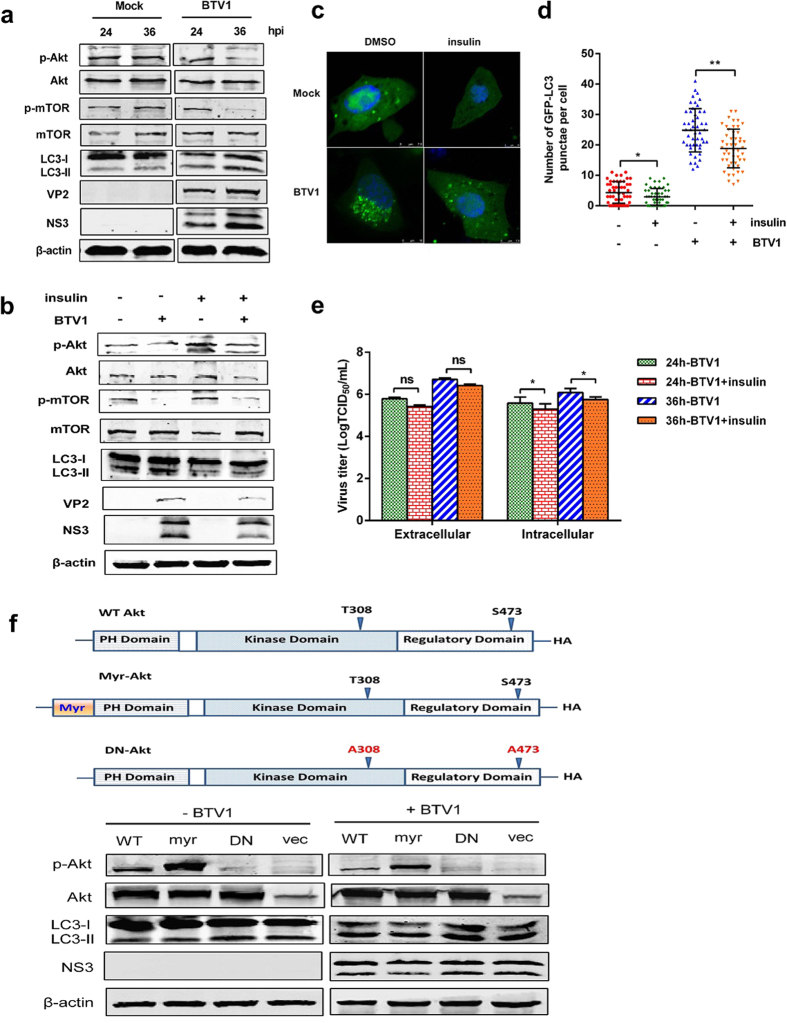
Akt inactivation contributes to inhibition of p-mTOR after BTV1 infection. (**a**) Western blotting analysis of BSR cells with BTV1 infection or mock infection for 24 and 36 h. Blots were analyzed with antibodies against p-Akt, Akt, p-mTOR, mTOR, VP2, NS3 and β-actin. (**b**) Effects of insulin treatment on autophagy level and mTOR activity. BSR cells were exposed to mock-infection or BTV1-infection in the presence or absence of 100 μg/mL insulin for 36 h and processed by immunoblotting using the corresponding antibodies. (**c**,**d**) BSR cells transfected with EGFP-LC3 plasmid were treated as in (**b**). Green punctae visualized by immunofluorescence (scale bar: 7.5 or 10 μm). The average number of green punctae per cell in each treatment was recorded. (**e**) Effects of 100 μg/mL insulin on the replication of BTV1. The extracellular and intracellular viral titers were determined by TCID_50_ assay at 24 hpi and 36 hpi, respectively. *P < 0.05, compared to control. (**f**) Western blotting analysis of the effects of Akt mutants on autophagy in BSR cells infected with BTV1. BSR cells were transfected with empty vector (vec), plasmids encoding wildtype Akt (WT-Akt, WT), constitutively active Akt (myr-Akt, myr), or dominant-negative Akt (DN-Akt, DN). After 12 h, cells were infected with BTV1 for 36 h. Blots were analyzed with antibodies against the indicated proteins.

It has been shown that mTOR is directly linked to Akt, a downstream product of PI3K^36^, and the PI3K/Akt/mTOR signaling pathway plays an important role in cell autophagy. Therefore, we examined Akt activity to investigate whether autophagy induced by BTV1 infection was involved in this mTOR-dependent pathway. We found that BTV1 induced a marked reduction in Akt and mTOR activity as well as a great increase in LC3-II, especially at 36 hpi (Fig. 3a), implying that Akt positively regulates mTOR. Next, to further obtain evidence for the interconnection between decreased Akt signaling and mTOR-dependent autophagy, activator insulin was exposed to BSR cells with or without BTV1 infection. Figure 3b showed that after insulin treatment, the decreases in Akt and mTOR phosphorylation were restored in BTV1-infected or mock-treated cells, which led to reduced autophagy (Fig. 3b). Similar findings were seen in the confocal image of BTV1 plus insulin, showing fewer accumulated GFP-LC3 punctae in Fig. 3c,d. As expected, autophagy suppression led to decreased extracellular and intracellular virus yields with insulin treatment, especially at 36 hpi (Fig. 3e), indicating that Akt links to autophagy and BTV replication at a late stage of infection. Moreover, we constructed mutant plasmids to examine the effects of dominant-negative (DN) and constitutively active (myr) Akt variants on autophagy and virus replication. We found that the abundance levels of LC3-II and NS3 protein were partially recovered by the presence of DN-Akt and partially degraded by myr-Akt compared with the vector group or WT-Akt group (Fig. 3f). Taken together, our data show that the Akt-mTOR pathway contributes to the regulation of autophagy and naturally affects BTV1 infection. However, the effect of this signaling is not strongly influential on BTV1-conferred autophagy and virus replication, suggesting that other mechanisms may exist.

### CaMKKβ-AMPK axis may be involved in BTV1-induced autophagy

**Figure 4:**
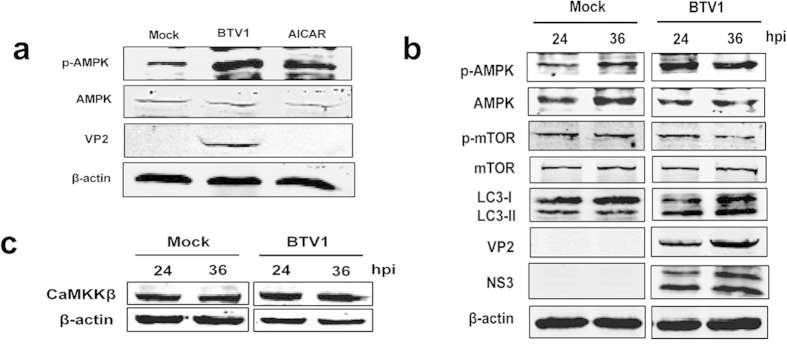
Activated AMPK may be involved in BTV1-induced autophagy and related to CaMKKβ. (**a**) Immunoblotting of p-AMPK and total AMPK in BSR cells infected with BTV1 or mock-infected or treated with AICAR (positive control) for 36 h. (**b**) BSR cells were infected as above. At different time points (24 and 36 h) post-infection, cells were harvested and analyzed the activity of AMPK, mTOR and LC3 by western blotting using the indicated antibodies. (**c**) Western blotting analysis of the effect of BTV1 infection on the extent of CaMKKβ.

In addition to Akt, another key mediator of mTOR is AMPK, which has been demonstrated to negatively regulate mTOR activity^37^,^38^. We sought to determine whether BTV-induced mTOR inactivation in BSR cells involves AMPK. First, BSR cells were infected with BTV1 or treated with AICAR (an AMPK activator)^39^ for 36 h to assess the AMPK activity. Figure 4a shows that BTV1 infection led to increased AMPK activity in BSR cells, as evidenced by the heightened expression level of phospho-AMPK. Then, we further analyzed the links of AMPK activation and mTOR inhibition with BTV1 infection. Figure 4b shows that BTV1-infection significantly increased the phosphorylation of AMPK at 24 and 36 hpi, while the levels of p-mTOR steadily decreased, which accordingly resulted in an increase in LC3-II. Thus, it is reasonable to speculate that AMPK might relate to the mTOR signaling pathways.

Previous reports proposed that AMPK could be activated via Ca^2+^/calmodulin-dependent protein kinase kinase (CaMKKβ) and subsequently inhibited mTOR^40^. So the level of CaMKKβ was also examined and found elevated in BTV1-infected cells at 24 and 36 hpi compared with mock cells (Fig. 4c). This observation is consistent with the result for AMPK. Accordingly, it is reasonable to speculate that the CaMKKβ-AMPK-mTOR signaling pathwaymay be involved in BTV1-induced autophagy.

### AMPK is authentically an upstream regulator of mTOR, and CaMKKβ is responsible for the AMPK-mTOR axis

Based on the aforementioned speculation, we further collected evidence to support this viewpoint. First, the role of AMPK in BTV1-reduced autophagy was verified.

**Figure 5:**
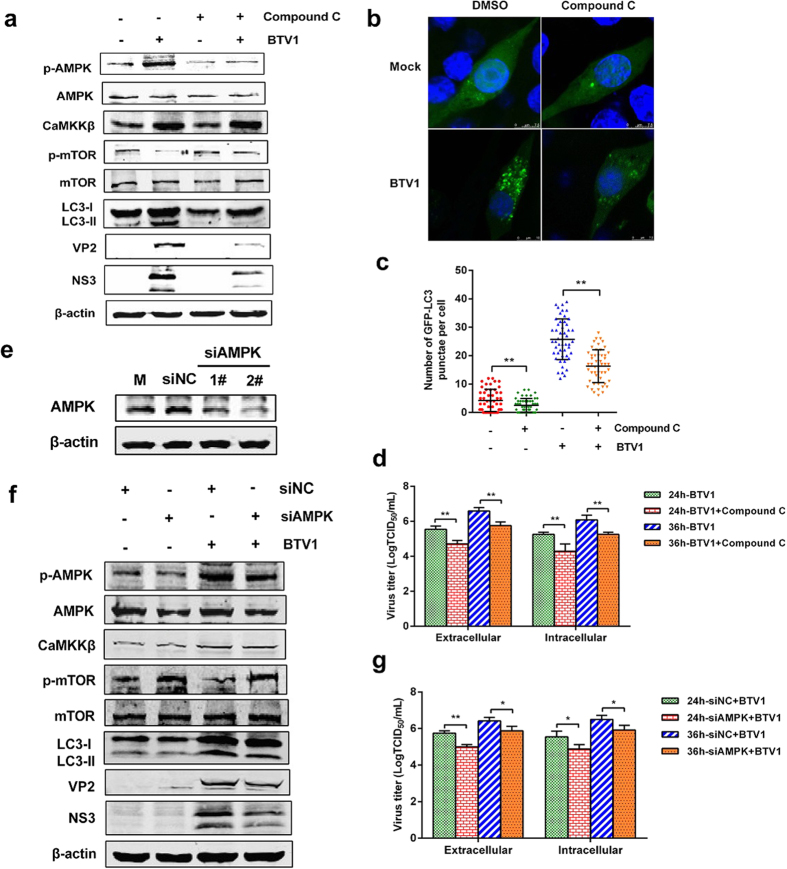
AMPK is assuredly an upstream regulator of mTOR in BTV1-induced autophagy. (**a**) Effects of Compound C treatment on LC3-II, CaMKKβ, phosphorylation and total levels of AMPK and mTOR. Cells were pretreated with Compound C (5 μM) or DMSO (Control) for 1 h, followed by BTV1 adsorption for 1 h. Then, the cells were cultured in the absence or presence of Compound C (5 μM). At 36 hpi, the protein levels were measured by western blotting. (**b**,**c**) BSR cells transfected with pEGFP-LC3 for 12 h were treated with 5 μM Compound C or BTV1, as before. The formation of GFP-LC3 punctae was analyzed, and the average number of green punctae per cell in each treatment was counted. (**d**) Titers of BTV1 produced by Compound C-treated BSR cells. Cells were pretreated and infected as described in (**a**). Extracellular and intracellular virus yields are shown as TCID_50_/mL at 24 hpi and 36 hpi. **P < 0.01, significantly different. (**e**) Western blotting analysis of the effectiveness of AMPK knockdown in BSR cells. BSR cells were transfected with control siRNA (siNC) or AMPK-specific siRNA for 36 h and then analyzed by Western blotting. (**f**) Knockdown of AMPK affects the activity of mTOR and autophagy in BTV1-infected cells. BSR cells were transfected with either control siRNA or AMPK-specific siRNA, then infected with BTV1. At 36 hpi, cells were harvested, and western blotting was performed. (**g**) Extracellular and intracellular virus yields in BSR cells transfected with siRNA against AMPK. Data are shown as TCID_50_/mL at 24 hpi and 36 hpi. *P < 0.05, **P < 0.01.

Because AMPK is activated with BTV1 infection, Compound C, an agent known to inhibit AMPK^41^, was used to assess the alteration of p-mTOR and CaMKKβ corresponding to AMPK inhibition. Figure 5a shows that in BTV1-infected cells, AMPK inhibition partly restored the phosphorylation of mTOR, and the restoration of mTOR activity subsequently inhibited BTV1-induced autophagy, whereas the inhibition of AMPK by Compound C did not inhibit CaMKKβ activation in response to BTV1 infection, suggesting that AMPK is a negative regulator of mTOR and occurs downstream of CaMKKβ. The puncta formation of GFP-LC3 was further examined. Compared with uninfected cells, significant puncta formation was found in BTV-infected cells but was dramatically reduced with the application of Compound C (Fig. 5b,c), so we concluded that AMPK could positively regulate autophagy. Additionally, in our previous study, we found that autophagy affected the state of BTV infection. Here, we found that the inhibition of autophagy with Compound C via AMPK not only downregulated the expression of VP2 and NS3 in BTV1-infected cells at 36 hpi (Fig. 5a) but also decreased the yields of BTV progeny both inside and outside the infected cells at 24 and 36 hpi (Fig. 5d). Our findings are consistent with previous observations.

To exclude any non-specific effects of the pharmacological experiment, RNA interference was used to suppress the expression of AMPK. Two pairs of siRNAs targeting AMPK were designed, and the silencing effect was tested (Fig. 5e). The effective mixture of those siRNAs targeting AMPK were used. As shown in Fig. 5f, compared with negative control siRNA (siNC)-transfected cells, BSR cells transfected with AMPK-targeting siRNAs exhibited a notable increase in p-mTOR protein but no obvious change in CaMKKβ. LC3-II formation was also greatly reduced by the suppression of AMPK expression, accompanied by significantly reduced viral protein synthesis. Meanwhile, the extracellular and intracellular virus particle yields also showed obvious reductions at 24 and 36 hpi (Fig. 5g). These results were consistent with the pharmacological studies. Taken together, our data suggest that AMPK is authentically an upstream regulator of mTOR and is required for the effective replication of BTV.

**Figure 6:**
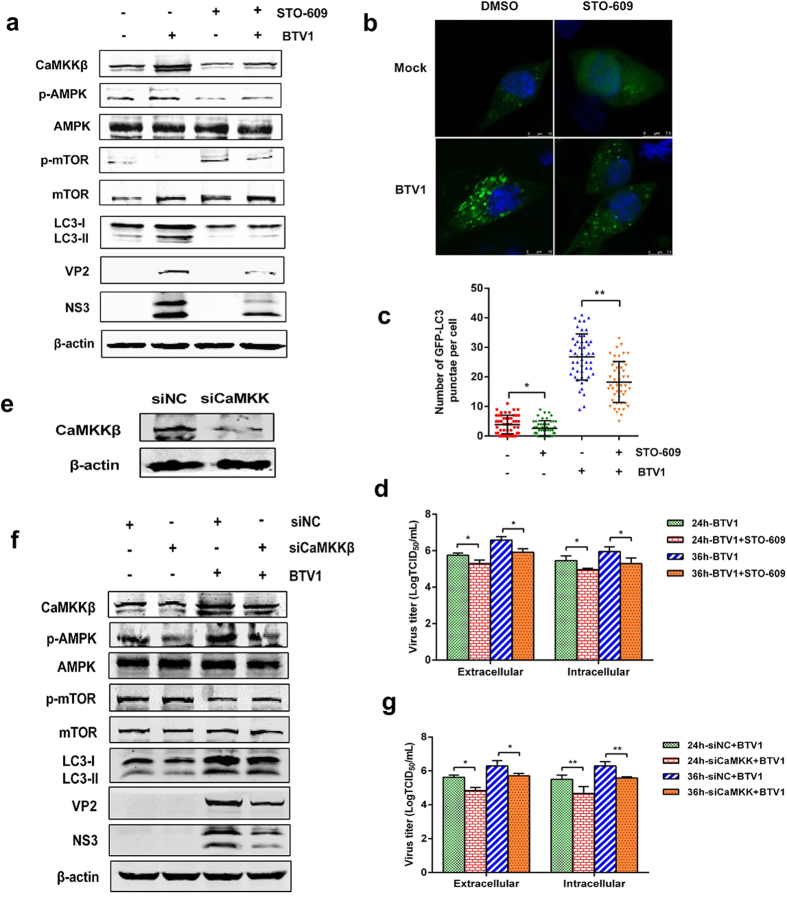
CaMKKβ is responsible for AMPK-mTOR axis in BTV1-induced autophagy. (**a**) Inhibition of CaMKKβ by STO-609 disturbed AMPK-involved autophagy. BSR cells with 50 μM STO-609 treatment or not infected with BTV1 or mock infected for 36 h, with detection of the levels of CaMKKβ, p-AMPK, p-mTOR and LC3-II by Western blotting. Mock cells were used as the control. (**b**,**c**) LC3 punctae were affected by STO-609. BSR cells transfected with pEGFP-LC3 for 12 h were treated with 50 μM STO-609 or BTV1 as before. The formation of GFP-LC3 punctae was analyzed, and the average number of green punctae per cell in each treatment was counted. (**d**) Both the extracellular and intracellular virus titers were measured with STO-609 treatment or the lack thereof in BTV1-infected cells at 24 hpi and 36 hpi. Data are shown as TCID_50_/mL. *P < 0.05, significantly different. (**e**) The silencing effectiveness of CaMKKβ in BSR cells was evaluated by Western blotting. BSR cells were transfected with negative control siRNA or specific siRNA against CaMKKβ for 36 h and then analyzed by Western blotting. (**f**) Knockdown of CaMKKβ affects the AMPK-mTOR axis and autophagy in BTV1-infected cells. BSR cells were transfected with either siNC or CaMKKβ-specific siRNA for 12 h, then infected with BTV1 or mock infected for an additional 36 h, after which cells were harvested for western blotting assay. (**g**) The extracellular and intracellular virus yields in BSR cells transfected with siRNA against CaMKKβ. Data are shown as TCID_50_/mL at 24 hpi and 36 hpi. *P < 0.05, **P < 0.01.

Next, the role of CaMKKβ in the AMPK-mTOR axis was checked. BSR cells were infected with BTV1, as previously performed, in the absence or presence of STO-609, a specific CaMKKβ inhibitor^42^. Lysed samples were used to evaluate the activity of AMPK and mTOR. As expected, STO-609 treatment not only significantly inhibited the BTV-induced increase in the phosphorylation of AMPK but also restored the BTV-induced decrease in the phosphorylation of mTOR, implying that CaMKKβ is an upstream regulator of the AMPK-mTOR axis (Fig. 6a). A large number of GFP-LC3 puncta were observed in BTV1-infected cells, an indicator of autophagy activation, whereas these puncta were reduced dramatically upon STO-609 treatment, suggesting that activated autophagy was inhibited (Fig. 6b,c). Thus, we concluded that CaMKKβ could positively regulate autophagy. The significant reduction in extracellular viral load and intracellular virus titer when exposed to STO-609 reflected that CaMKKβ is required for BTV1 replication (Fig. 6d).

As before, siRNAs targeting CaMKKβ were used to confirm the results of the drug tests (Fig. 6e). As shown in Fig. 6f, compared with the negative control group, BSR cells transfected with CaMKKβ-targeting siRNAs exhibited a similar phenomenon with STO-609 treatment: AMPK expression and LC3-II level were suppressed, and p-mTOR protein was regained. The depressed extracellular and intracellular viral loads (Fig. 6g) and reduced viral proteins synthesis (Fig. 6f) indicated that autophagy inhibition via CaMKKβ inhibition also disturbed BTV1 replication. In brief, our data suggest that CaMKKβ is responsible for the AMPK-mTOR axis and BTV1 infection.

### BTV1-induced [Ca^2+^]_cyto_ increase serves as a potent inducer of autophagy through CaMKKβ and AMPK

In an attempt to elucidate further the mechanism, upstream inducer was progressively explored. It is now well established that intracellular Ca^2+^ is one of the regulators of autophagy and plays a dual role^43^. Some reports indicated a stimulatory role for Ca^2+^ towards autophagy, that is to say, elevated cytosolic Ca^2+^ concentration ([Ca^2+^]_cyto_) would promote the autophagic process^40^,^44^. More importantly, a previous study indicated that the elevated [Ca^2+^]_cyto_-mediated induction of autophagy is proposed to occur through the activation of CaMKKβ, which phosphorylates and activates AMPK^40^. Thus, we next explored the status of [Ca^2+^]_cyto_, a possible upstream regulator of CaMKKβ and AMPK, in BTV1-infected cells.

**Figure 7:**
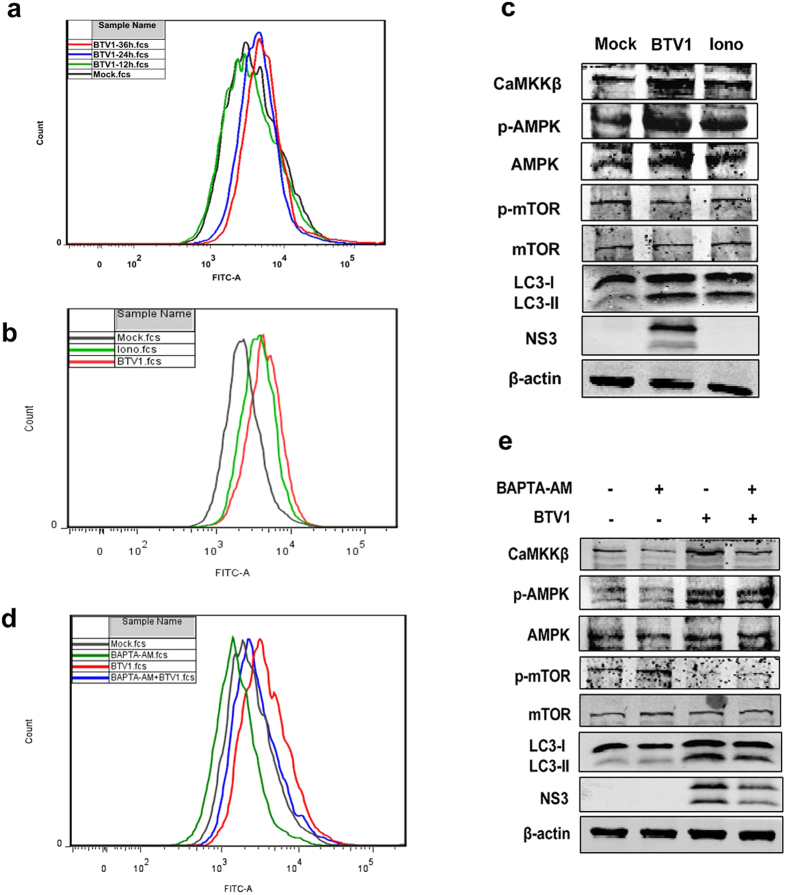
[Ca^2+^]_Cyto_ level affects autophagy through CaMKKβ and AMPK. (**a**) Effects of BTV1 infection on intracellular Ca^2+^ level in BSR cells. The BSR cells infected with BTV1 at the indicated times were incubated with Fluo-3 AM as a probe and the intracellular Ca^2+^ was measured using a flow cytometer. The shifts of fluorescence peaks were recorded. (**b**) BSR cells were left untreated (Mock) or treated with BTV1 (MOI = 1) or 4 μM ionomycin (Iono, positive control) for 30 h. Then the intracellular Ca^2+^ was measured using a flow cytometer as described above. (**c**) Proteins from BSR cells left untreated (Mock) or treated with BTV1 or 4 μM ionomycin (Iono, positive control) for 30 h were analyzed by immunoblotting for the activities of CaMKKβ, AMPK , mTOR and LC3. (**d**) After BTV1 adsorption for 1 h, BSR cells were cultured in the absence or presence of BAPTA-AM (25 μM). At 30 hpi, [Ca^2+^]_Cyto_ level were analyzed as in (**b**,**e**) The levels of related proteins were detected by Western blotting after mock-infection or BTV1-infection in the presence or absence of 25 μM BAPTA-AM for 30 h.

The BSR cells infected with BTV1 at the indicated times were incubated with Fluo-3 AM, and intracellular Ca^2+^ was monitored using a flow cytometer. We observed that the fluorescence intensity of the peaks shifted rightward with the infection, meaning that [Ca^2+^]_cyto_ levels increased progressively with increasing infection time (Fig. 7a). Further, to determine whether the BTV-induced increase in [Ca^2+^]_cyto_ actually performs signaling through CaMKKβ and AMPK for autophagy induction, we first applied ionomycin as a positive control to perform our assay. Ionomycin (Iono), a Ca^2+^ ionophore that serves as a mobile Ca^2+^ carrier, allows Ca^2+^ fluxes from both the extracelluar space and the ER causing a rapid burst in cytosolic calcium levels and CaMKKβ and AMPK activation^45^. As shown in Fig. 7b,c, compared with mock cells, BSR cells treated with BTV1 or ionomycin exhibited similar change trends in [Ca^2+^]_cyto_ and related moleculars, suggesting that BTV-induced Ca^2+^ signaling may lead to autophagy stimulation by the same way. Based on these findings, we reasoned that if chelation of calcium resulted in contrary effects. 1,2-Bis(2-aminophenoxy)ethane-N,N,N′,N′-tetraacetic acid tetrakis(acetoxymethyl ester) (BAPTA-AM) is a well-established chelator of intracellular calcium ions^40^. As expected, the fluorescence intensity of the peak shifted leftward with BAPTA-AM treatment, indicating that BAPTA-AM treatment indeed obviously reduced [Ca^2+^]_cyto_ level (Fig. 7d). Accordingly, the addition of BAPTA-AM to the cells significantly abolished the activities of CaMKKβ and AMPK. In addition, the remarkable decrease of LC3 expression with BAPTA-AM was accompanied by an obvious reduction in viral proteins synthesis (Fig. 7e). All these reflected that Ca^2+^ is required for functional autophagy in a CaMKKβ- and AMPK-dependent manner during BTV infection.

### TSC2 is intermediate of Akt/AMPK-mTOR signaling pathways

**Figure 8:**
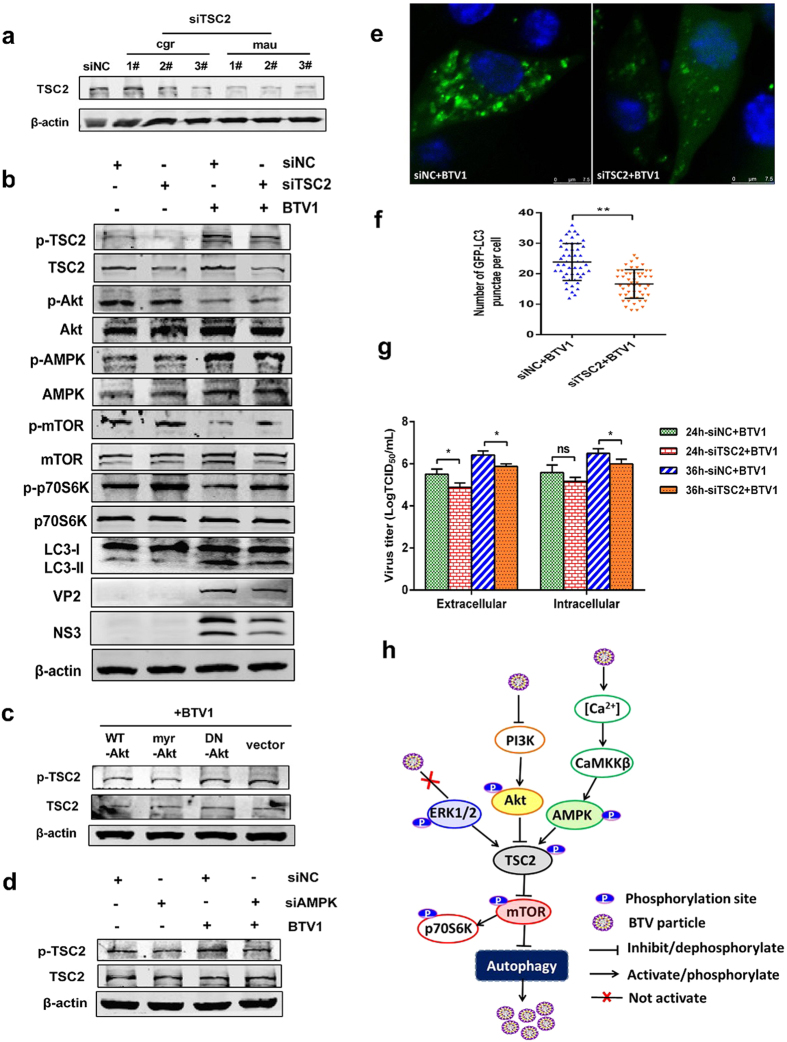
TSC2 is an intermediate of Akt-mTOR and AMPK-mTOR signaling pathways. (**a**) Western blotting analysis of the effectiveness of TSC2 knockdown in BSR cells: siRNAs targeting TSC2 (*Mesocricetus auratus*/mau) and TSC2 (*Cricetulus griseus*/cgr) (siTSC2) were screened and analyzed by Western blotting, and siNC was used as a control. (**b**) Effects of TSC2 silencing on the signaling molecules in autophagy. BSR cells were transfected with TSC2 siRNA or siNC for 12 h, followed by mock infection or BTV1 infection. At 36 hpi, related proteins were analyzed by western blot. (**c**) Determination of the effects of Akt mutants on TSC2 activity. BSR cells were transfected with empty vector (vec), plasmids encoding wildtype Akt (WT-Akt), constitutively active Akt (myr-Akt), or dominant-negative Akt (DN-Akt). After 12 h, cells were infected with BTV1 for 36 h. TSC2 activity was assessed through blots. (**d**) Knockdown of AMPK affects the activity of TSC2. BSR cells were transfected with siNC or siAMPK for 12 h, and cells were then infected with BTV1. At 36 hpi, cells were harvested, and western blotting was performed to examine TSC2 activity. (**e**,**f**) Representative confocal images of BTV with or without siTSC2 treatment for 36 h. BSR cells were transfected with pEGFP-LC3, along with siTSC2 or siNC. At 12 hpt, cells were treated with BTV1 for another 36 h. GFP-LC3 punctae were analyzed, and the average number of green punctae per cell in each treatment was counted. (**g**) The extracellular and intracellular BTV1 yields produced by siNC or siTSC2-transfected BSR cells were tested and shown as TCID_50_/mL at 24 hpi and 36 hpi. *P < 0.05, significantly different. (**h**) A hypothetical model signaling network involving ERK1/2, Akt and AMPK in BTV1-induced autophagy. BTV1 infection induces the suppression of Akt and upregulation of AMPK via [Ca^2+^]_cyto_-mediated CaMKKβ activation, leading to the TSC2-mediated inhibition of mTOR and downstream p70S6K, ultimately leading to autophagy initiation. In addition, ERK1/2 is not involved in BTV1-induced autophagy. The whole signaling network is utilized by BTV1 for its own benefit.

It is clear that the activity of mTORC1 is negatively regulated by the tuberous sclerosis 1/2 (TSC1/TSC2) complex, and TSC2 can be positively regulated by AMPK or negatively regulated by Akt^28^,^46^. Based on our existing data, we then planned to focus our attention on TSC2. To address whether TSC2 is an essential intermediate in both the Akt-mTOR and AMPK-mTOR signaling pathways, TSC2 gene knockdown was first performed using TSC2-specific siRNAs to predict related molecules. Some pairs of siRNAs targeting TSC2 (*Mesocricetus auratus*/mau, GI:524963743) and TSC2 (*Cricetulus griseus*/cgr, GI:625196261) were designed and screened, and we chose siTSC2 (mau2#) for further use because it exhibited the most significant effect (Fig. 8a). Nontarget siRNA was used as a negative control (siNC). We found that BTV1 infection induced the activation of TSC2 and reduced mTOR activity. The suppression of TSC2 by siRNA recovered mTOR phosphorylation and then downregulated LC3-II (Fig. 8b), suggesting that TSC2 regulated autophagy via mTOR. Furthermore, Akt inhibition and AMPK activation caused by BTV1 infection were not markedly altered in BSR cells treated with TSC2-specific siRNA compared to cells treated with control siRNA (Fig. 8b), implying that Akt and AMPK might be upstream regulators of TSC2 in BTV1-mediated autophagy. To thoroughly confirm this conclusion, we transfected BSR cells with Akt mutant plasmids and siRNAs against AMPK, respectively, then infected the cells with BTV1 for 36 h, and the protein lysates were analyzed. We found that the activation of Akt with plasmid myr-Akt repressed p-TSC2, and the inhibition of Akt with DN-Akt recovered TSC2 activity (Fig. 8c). The inhibition of AMPK also suppressed TSC2 activation (Fig. 8d). All these results indicated that TSC2 is a crucial downstream effector of Akt and AMPK. In addition, LC3 aggregation was markedly decreased in infected cells treated with TSC2-specific siRNA compared to cells treated with control siRNA (Fig. 8e,f), which suggested that TSC2 was required for the BTV1-induced autophagic response. TSC2 interference ultimately led to impaired replication of BTV through the disturbance of autophagy (Fig. 8g). These results imply that TSC2 is a pivotal intermediate of the Akt-mTOR and AMPK-mTOR signaling pathways, ultimately contributing to BTV1-induced autophagy. Figure 8h shows a hypothetical model depicting the signaling mechanisms of BTV1-induced autophagy in BSR cells.

### Pharmacological treatments and siRNA transfection minimally affect cell viability

To assess whether pharmacological treatments and siRNA transfection affected cell viability, which would ultimately affect the proliferation of the virus in host cells, we used the WST-1 (updated substitution of MTT) assay to analyze their effects on cell viability. Based on the results, we conclude that chemical application and siRNA transfection exhibited almost no significant effects on cell viability (See supplementary Fig. S1).

## Discussion

Autophagy is a tightly regulated and evolutionarily conserved cellular process in which cells destroy and recycle their own components in lysosomes. Accumulating lines of evidence have suggested that many virus-induced autophagies play important roles in the viral life cycles and pathogenesis^47^. Several viruses have been shown to evade, subvert, or exploit autophagy, seemingly to insure their own replication or survival advantages, including rotavirus, poliovirus, enterovirus 71, coronavirus, Epstein-Barr virus (EBV), hepatitis C virus (HCV), and hepatitis B virus (HBV)^10^. In our previous work, we found that BTV, a complex dsRNA virus belonging to the *Reoviridae* family, also triggered autophagy for its own benefit in host cells^14^. However, the exact mechanism activated by BTV to initiate the autophagy process remains largely unknown. In this study, we broadly describe a signaling cascade network induced by BTV1, involving ERK1/2, Akt and AMPK, integrated by mTOR, resulting in the autophagy response to provide a favorable cellular environment for the viral needs (Fig. 8h).

Many signaling cascades have been implicated in regulating autophagy, in response to different intra- or extracellular stimuli. Similarly to yeast, the classical pathway involves the well-known serine/threonine kinase mTOR, a master regulator. Various pathways and small molecules regulate autophagy via mTOR or mTOR-independent mechanisms, which have been identified in recent years^17^. The mTOR pathway involves two functional complexes: the rapamycin-sensitive mTOR complex 1 (mTORC1), which regulates autophagy, and mTOR complex 2 (mTORC2), which is not a direct autophagy regulator. The activity of mTOR is controlled by many upstream signals, e.g., PI3K/Akt, AMPK and p53, which subsequently regulate related down-stream molecules and initiate the autophagy process. We present our current knowledge regarding the possible key genes composing the autophagy machinery induced by BTV infection.

The mitogen-activated protein kinase (MAPK)/extracellular signal-regulated kinase (ERK) pathway, which is commonly activated in cancers, regulates autophagy^15^. ERK1/2 has recently been shown to inhibit mTOR via activating TSC2, thus enhancing Beclin1-mediated autophagy^48^. ERK1/2 signaling has been observed in porcine circovirus type 2 and avian reovirus, which both regulate autophagy^33^,^34^. However, in our observations, ERK1/2 is not involved in the autophagy induced by BTV1 infection (Fig. 2).

A major signaling cascade controlling mTORC1 is the PI3K pathway. Akt has been shown to modulate the autophagic activity regulated by the PI3K pathway. The different classes of PI3K exert distinct effects on autophagy^49^. Increases in the class I PI3K product, PIP3, inhibit autophagy by inducing Akt activation. In other words, class I PI3K/Akt is a negatively regulated pathway of autophagy. In contrast, increasing the levels of the class III PI3K product, PI-3-P, stimulates autophagy. For BTV1, our results suggest that the down-regulation of p-mTOR can be partly attributed to Akt inactivation after BTV1 infection, which leads to autophagy activation (Fig. 3). Mutations causing Akt activation or inactivation cause the suppression and induction of autophagy, respectively. These results are consistent with a previous report^50^. Similarly to BTV, with slight differences, avian reovirus protein p17-mediated Tpr suppression negatively regulates PI3K/Akt/mTOR signaling for its own replication, and HCV infection induces ER stress, which inhibits the Akt-TSC-mTORC1 pathway, thus inhibiting ULK1, and subsequently upregulates autophagy^34^,^51^. Thus, we deduce that different viruses utilize the PI3K/Akt/mTOR pathway to promote autophagy through various stimulatory molecules. In addition, it is worth mentioning that the functions of the PI3K/Akt pathway are not limited to autophagy induction. The PI3K/Akt signaling pathway is involved in multi-cellular processes such as glucose metabolism, protein synthesis, and proliferation. It also appears to be associated with the host cell immune response to counteract viral infection^52^. Moreover, previous investigations have suggested that many viruses rely on the activation of this pathway for their replication^53^,^54^. Hence, we conclude that the PI3K/Akt pathway is a complex and crucial pathway that is distinctively manipulated by various viruses, depending on the virus type or stage of viral replication.

AMPK is a critical regulator of the cellular metabolism and plays an important role in the autophagy process. Phosphorylated AMPK can either negatively regulate the mTOR complex (mTORC1) or directly phosphorylate ULK1, both of which can initiate the autophagy process^38^. Tracking back upstream from AMPK demonstrated that two signals were involved, Ca^2+^-mediated CaMKKβ and cellular energy. Calcium and CAMKKβ are involved in AMPK activation in hypothalamic neurons, T cells and endothelial cells, indicating that the calcium metabolism also plays a role in AMPK-mTOR-mediated autophagy regulation^40^. Recent research findings have shown that, in rotavirus-infected cells, CaMKKβ is stimulated by the increase in the intracellular Ca^2+^ level and further activates AMPK, the latter potently inducing autophagy^55^. Rotavirus-encoded viroporin NSP4 plays a key role in this process. Because BTV and rotavirus all belong to the *Reoviridae* family, and the NS3 of BTV shares a similar structure with viroporin, this CaMKKβ signaling was verified in BTV1-induced autophagy. Quite predictably, BTV infection caused the elevation of [Ca^2+^]_cyto_ and Ca^2+^-mediated activation of CaMKKβ, leading to the phosphorylation of AMPK and the initiation of autophagy (Figs 4, 5, 6, 7). Although BTV1 infection indeed could elevate cytosolic calcium, the mechanisms by which this occurs are unclear. We speculated that BTV might encode one or more proteins to change the biomembrane permeability to calcium, causing the increased [Ca^2+^]_cyto_ originated from ER or Golgi [Ca^2+^] stores, or extracellular environment. In our previous study, we have confirmed that ER stress response is involved in BTV-induced autophagy^56^, which remined us there might be some links between ER stress and elevated [Ca^2+^]_cyto_. It’s worth noting that many viruses, such as poliovirus, coxsackievirus, and hepatitis B virus, all could induce [Ca^2+^]_cyto_ increase as well as CaMKKβ activation^57^,^58^, leading to the speculation that virus-mediated CaMKKβ activation through elevated [Ca^2+^]_cyto_ might be a common mechanism for the induction of autophagy. Moreover, we noted that the effects of AMPK inhibition on BTV1-induced autophagy are slightly greater than the effects of CaMKKβ inhibition, so we speculated that AMPK might be simultaneously activated by some other upstream signals, which are currently unclear. According to the literature reports, AMPK is a serine-threonine kinase that functions as a fuel gauge and maintains energy homeostasis during cellular stress^59^. AMPK is sensitive to the cytosolic AMP-to-ATP ratio, and metabolic stress activates autophagy through the suppression of mTOR signaling^24^. Thus, whether the decreased ATP level is triggered by BTV1 infection and participates in the activation of AMPK and the induction of autophagy needs to be further explored.

In conclusion, our findings suggest that BTV1-induced autophagy is initiated by repressing mTOR through Akt inhibition and AMPK activation and is independent of ERK1/2 signaling. All these autophagic pathways may be utilized to sustain and favor BTV1 replication. Clearly, our understanding of the molecular mechanisms remains insufficient, as several aspects of BTV-induced autophagy remain unclear. The crosstalk or linkage between these signaling pathways that act in controlling autophagy needs to be further explored, and detailed studies on potential new pathways should be conducted.

In summary, our data presented here identify a crucial step in driving the progress of interplay between BTV and autophagy. Our current knowledge on the signaling regulations of BTV-induced autophagy will provide new insights into the pathogenesis of BTV and contribute to potential antiviral drugs development.

## Methods

### Cells and viruses

BSR cells (baby hamster kidney cell clones) were purchased from ATCC and cultured in Dulbecco’s modified Eagle’s medium (DMEM, Invitrogen, Carlsbad, CA, USA) supplemented with 10% fetal bovine serum (FBS, Gibco, Carlsbad, CA, USA), 100 U/ml penicillin, and 100 μg/ml streptomycin at 37 °C in a 5% CO_2_ humidified atmosphere. BTV1 strain SZ97/1 (GenBank accession numbers JN848759 to JN848768) was propagated in BSR cells.

### Plasmids, antibodies, and reagents

To construct pEGFP-LC3B, the LC3B gene was amplified from BSR cells with primers (LC3F:GTGAATTCTCCGTCCGAGAAGACCTTCAAG; LC3R:AAGTCGACTTACACAGCCATTGCTGTCCCGA) that were designed based on the sequence of LC3B (GenBank No. NM_026160.4) and then cloned into pEGFP-C1 to express LC3B fused with the EGFP protein at its N-terminus.

The mouse Akt cDNA clone was obtained from OriGene. In addition to the wild type Akt, plasmids encoding dominant negative and constitutively active Akt variants were constructed by site-directed mutagenesis (T308A and S473A) or myr tag introduction (ATGGGGAGCAGCAAGAGCAAGCCCAAGTCTAGA). All the cDNAs of Akt mutants were cloned into the pCAGGS-HA (pHA) vector. The primers used in this study are available upon request. All constructs were validated by DNA sequencing.

Primary antibodies against p-mTOR(Ser2448), mTOR, p-p70S6K(Thr389), p70S6K, p-ERK1/2 (Thr202/Tyr204), p-Akt(Ser473), Akt, p-AMPKα(Thr172), and AMPKα were purchased from Cell Signaling Technology (Danvers, MA, USA). CaMKKβ antibody was purchased from Pierce (Thermo Fisher Scientific, Rockford, IL, USA). Rabbit anti-LC3B antibody was purchased from Sigma-Aldrich (St Louis, MO, USA). Antibody against TSC2 [tuberin (C-20)] was purchased from Santa Cruz Biotechnology (Dallas, TX, USA). Anti-Tuberin (phospho T1462) antibody was obtained from Abcam (Cambridge, CB4 0FL, UK). Anti-β-actin antibody was purchased from Zhong Shan-Golden Bridge Biotechnology (Beijing, China), Mouse monoclonal antibodies against the VP2 and NS3 proteins of BTV1 were prepared by our laboratory. IRDye 800 CW goat anti-mouse IgG or goat anti-rabbit IgG, as secondary antibodies, were purchased from LiCor BioSciences (Lincoln, NA, USA).

Rapamycin (Rapa), U0126, insulin, AICAR, Fluo-3 AM, Ionomycin and WST-1 cell proliferation and cytotoxicity assay kits were purchased from Beyotime (Beijing, China). Compound C was purchased from Calbiochem (Merck-Millipore, Darmstadt, Germany). STO-609 was purchased from Tocris Bioscience (R&D Systems, Missouri, UK). BAPTA-AM was purchased from Abcam (Cambridge, CB4 0FL, UK).

### Chemicals treatments and virus infection

Chemicals and their optimal concentrations used in this experiment included 100 nM rapamycin, 20 μM U0126, 100 μg/mL insulin, 330 μM AICAR, 5 μM Compound C, 50 μM STO-609, 4 μM ionomycin, and 25 μM BAPTA-AM. After pretreatment, BSR cells were infected with BTV1 at a multiplicity of infection (MOI) of 1 for 1-hour absorption. The cells were then cultured in fresh media in the absence or presence of the same drug as pre-treatment for the indicated times. The corresponding solvent dimethyl sulfoxide (DMSO) or ddH_2_O was used as a control.

### Plasmid or small interfering RNA (siRNA) transfection

Akt variants were constructed to assess their effects on autophagy. BSR cells were grown to 70–80% confluence and then transiently transfected with wild type Akt (WT-Akt), myr-tagged constitutively active Akt (myr-Akt) and dominant negative (T308A and S473A) Akt (DN-Akt) using Lipofectamine 2000 reagent (Invitrogen, Carlsbad, CA, USA) according to the manufacturer’s instructions. At 12–18 hours post-transfection (hpt), the cells were subjected to different treatments. The empty pCAGGS vector was used as a control.

Small interfering RNAs (siRNAs) targeting AMPK, CaMKKβ and TSC2 were synthesized by GenePharma Co., Ltd. (Shanghai, China). To evaluate the efficiency of the siRNAs, targeted siRNAs and a negative control siRNA (siNC) were transfected into BSR cells cultured in a 12-well plate using Lipofectamine 2000, according to the manufacturer’s protocol. The knockdown effects were analyzed by Western blotting. After transfection with the most effective siRNAs, the cells were infected with BTV1 (MOI = 1) at 12 hpt and incubated for 24 h or 36 h before the samples were collected. The effective siRNA sequences were as follows: AMPK-1#, 5′-GCAUAUGCUGCAGGUAGAUTT-3′ (sense) and 5′-AUCUACCUGCAGCAUAUGCTT-3′ (antisense); AMPK-2#, 5′-CGUCAUUGAUGAUGAGGCUTT-3′ (sense) and 5′-AGCCUCAUCAUCAAUGACGTT-3′ (antisense); CaMKKβ, 5′-GCCAUGGGUGUGACACUAUTT-3′ (sense) and 5′-AUAGUGUCACACCCAUGGCTT-3′ (antisense); TSC2, 5′-CCUUCGUGCUGCUCAUUAATT-3′ (sense) and 5′-UUAAUGAGCAGCACGAAGGTT-3′ (antisense); negative control siRNA: 5′-UUCUCCGAACGUGUCACGUTT-3′ (sense) and 5′-ACGUGACACGUUCGGAGAATT-3′ (antisense).

### SDS-PAGE and Western blot

Following the indicated treatments, all samples were lysed using Western and IP lysis buffer (Beyotime, Beijing, China) containing PMSF and Complete Protease Inhibitor Cocktail (Roche, Penzberg, Germany). The clarified lysates were harvested and subjected to 12% SDS-PAGE gel, and the proteins were then electrotransferred to NC membranes. After blocking with 5% BSA for 2 h, the membranes were incubated with appropriate primary antibodies overnight at 4 °C, followed by IRDye 800 CW goat anti-mouse IgG or goat anti-rabbit IgG as secondary antibodies. The blots were visualized and quantified using an Odyssey Infrared Imaging System (LiCor BioSciences).

### Confocal fluorescence microscopy

BSR cells with 80% confluence in 35-mm glass-bottomed culture dishes (NEST) were transfected with pEGFP-LC3 using Lipofectamine 2000. At 12–18 hpt, the cells were treated with drugs or BTV1 as described above, then fixed with 4% paraformaldehyde at 24–36 h post-treatment. After permeabilization with 0.1% Triton X-100, the cell nucleus was counterstained with 4′, 6′-diamidino-2-phenylindole (DAPI). The EGFP-LC3 fluorescence dots were observed using a Zeiss LSM-510 laser scanning fluorescence microscope (Carl Zeiss AG, Jena, Germany). The average number of EGFP-LC3 punctae per cell from 50 cells per sample was counted^60^. Only cells with at least 5 dots were scored as positive.

### Virus titration assay

BSR cells in 6-well plates were treated with drugs or infected with BTV1 as previously described for the indicated times. Then, the extracellular viruses from the cell supernatants and the intracellular viruses from cells treated with multiple freeze-thaws were harvested at different times and serially diluted 10-fold to infect confluent BSR cells in 96-well plates. Five days post-infection, cytopathic effects (CPEs) were observed, and virus titers were calculated as TCID_50_/mL using the Reed-Muench method. All data are shown as the means of three independent experiments.

### Cell viability assay

The cell viability assay was performed using the WST-1 cell proliferation and cytotoxicity assay kit according to the manufacturer’s protocol. In brief, BSR cells were seeded in a 96-well plate at a density of 5000 per well and incubated at 37 °C for 24 h. The cells were treated with the drugs mentioned above at optimal concentrations or transfected with siRNAs, and the plates were incubated for 36 h. Then, 10 μL of WST-1 was added to each well, and the cells were further incubated for 2 h. The optical density at 450 nm was measured. The viability of the treated cells was expressed as a percentage relative to the untreated cells.

### Flow cytometry for intracellular calcium detection

The cytosolic free calcium was detected using Fluo-3 AM. Fluo-3 AM itself does not bind Ca^2+^, but it is readily hydrolyzed to fluo-3 once the dye is inside the cells, and fluo-3 will emit fluorescence only when it binds to Ca^2+^. The BSR cells infected with BTV1 or treated with ionomycin or BAPTA-AM for the indicated times were incubated with Fluo-3 AM at 37 °C for 1 h in darkness. After washing, the cells were resuspended in sterile phosphate buffered saline (PBS, pH 7.2), and the resulting fluorescence, as an indicator of intracellular Ca^2+^, was monitored using a flow cytometer (BD FACSAriaTM) at an excitation wavelength of 488 nm.

### Statistical Analysis

Data were presented as the mean ± standard deviation (SD) from three independent experiments. All statistical tests were performed using GraphPad Prism 6 (GraphPad Software, Inc., La Jolla, CA, USA). Statistical comparisons were performed using Student’s t test or ANOVA where appropriate. A *P* value of < 0.05 was considered statistically significant.

## Additional Information

**How to cite this article**: Lv, S. *et al.* Dissection and integration of the autophagy signaling network initiated by bluetongue virus infection: crucial candidates ERK1/2, Akt and AMPK. *Sci. Rep.* **6**, 23130; doi: 10.1038/srep23130 (2016).

## Supplementary Material

Supplementary Information
