# Supplementary material for: Dissection and integration of the autophagy signaling network initiated by bluetongue virus infection: crucial candidates ERK1/2, Akt and AMPK
Source: Sci Rep. 2016 Mar 15;6:23130. doi: 10.1038/srep23130 (PMC4791558; doi:10.1038/srep23130)

Supplemental Information for the Manuscript **"Dissection and integration of the autophagy signaling network initiated by bluetongue virus infection: crucial candidates ERK1/2, Akt and AMPK"**

Shuang Lv[1],Qing-Yuan Xu[1], En-Cheng Sun[1], Ji-Kai Zhang[1], Dong-Lai Wu[1]*

[1]State Key Laboratory of Veterinary Biotechnology, Harbin Veterinary Research Institute, Chinese Academy of Agricultural Sciences, Harbin 150001, China

Figure Legends

Supplemental Figure S1: Pharmacological or siRNA treatments do not affect cell viability.

Cell viability was detected by WST-1 assay after treatments with these drugs at the indicated concentrations or transfection with siRNAs in BSR cells for 36 h. Light absorption at 450 nm was recorded and expressed as a percentage of relative cell viability, and the values are represented as the mean±SD (n=3). Significant differences were assessed by one-way ANOVA. "ns" means no significant difference compared to control, $P > 0.05$.

Supplemental Figure S1

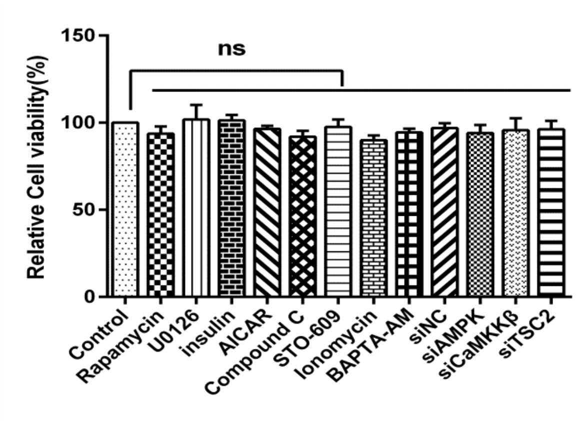

Supplement: Supplementary Information [file srep23130-s1.pdf]
